# A Stumping Experience: An Autobiographical Case Report

**DOI:** 10.7759/cureus.45625

**Published:** 2023-09-20

**Authors:** Dawood Sajid Dar

**Affiliations:** 1 Medicine, Shaikh Zayed Hospital, Lahore, PAK

**Keywords:** gastroenterology, general surgery, emergency, delayed complication, completion appendectomy, appendectomy, appendicitis, stump appendicitis

## Abstract

Stump appendicitis, while being well recognized as a delayed complication following an appendectomy, is a relatively under-reported clinical entity, with an even lesser documentation frequency in the subcontinent, from where the author hails. This article recounts his brief yet consequential journey from ill-health to recovery, all the while giving an insight into a multitude of related experiences that served as learning points for the narrator. Additionally, this account of events, having been viewed through a hybrid doctor-patient lens, aspires to be a valuable addition to medical literature.

## Introduction

Acute appendicitis is one of the most common surgical emergencies encountered in a healthcare setting, with an incidence of approximately 18 million cases in 2019 [1]. The standard treatment approach is appendectomy, either open or laparoscopic, which is generally considered to be a safe procedure with a low complication rate. Nevertheless, as with any other surgical intervention, complications can arise that can end up being a source of morbidity for the patient, adding to the existing physical and psychological stress caused by the primary illness.

Stump appendicitis, a potential postoperative complication following an appendectomy, can imperil the health of a patient if it is not diagnosed and managed promptly. This ailment is mimicked by an array of right iliac fossa pathologies and is hence often pushed lower down the differential list owing to a prior history of appendectomy, thus precluding it from being timely diagnosed. Furthermore, a low incidence frequency [2], along with it being an under-reported malady, results in it being less well-known among health professionals and enables it to pose as a diagnostic challenge, if and when it arises.

A rare illness, when not manifesting in its typical fashion, can prove to be a tricky diagnosis to reach. Such was the case in the author's experience, where the history and presenting symptoms did not seem to fit well with any definite clinical diagnosis. However, the author was fortunate enough to have access to specialists with sound clinical acumen, whose timely diagnosis and management enabled him to make a complete recovery from stump appendicitis, a complication that developed seven years after undergoing an open appendectomy in 2015. Additionally, this transition from a junior doctor to the role of a patient was a vexing yet enlightening experience, as it enabled the author to recognize the psychological impact a period of sickness can have on a person, a fact that is often overlooked by health professionals while being caught up in the demanding routine of an emotionally taxing job. Moreover, the author also came to realize the fact that a rare illness can manifest in an equally queer manner, that is, without its characteristic symptomatology. Thus, while it is imperative for physicians to be well equipped with knowledge about this condition, it is equally essential to adopt a holistic and multifaceted approach when dealing with any patient, which is one of the objectives this article hopes to achieve. Lastly, this case report aims to highlight the importance of "non-gold standard" modalities in clinical practice, as these can prove to be as useful and effective as first-line interventions.

## Case presentation

Back in 2015, a few weeks prior to embarking on my medical school journey, I was diagnosed with acute appendicitis, a condition relatively alien to me at that time. Although the only symptoms I developed were right lower quadrant pain, without its typical shifting character, and low-grade fever, the elicitable signs on examination and the ultrasonographic findings were unmistakable to the keen-eyed consultant. I vividly recall the dreaded trip to the hospital, the anxious wait while lying on the operating table as the paramedical staff prepared for surgery, and being out like a light seconds after being injected with "the milk of amnesia." The procedure, an open appendectomy, was carried out successfully and was followed by a minimal inhospital stay. The histopathological examination of the excised specimen evidenced no abnormalities apart from the expected signs of acute inflammation. Consequently, I was able to make a swift and complete recovery and was now ready to kickstart a new chapter of my educational career as a medical student.

During the subsequent five years, I was familiarized with the various aspects of the culprit organ, from learning about its intricate microscopic structure and its location in the body to understanding the process by which it gets caught up in a whirlwind of inflammation, which warrants a swift surgical response. After earning the coveted degree and a medical license, I started my first professional job as a house officer in a tertiary care hospital, during which I was made to transition to the role of a patient due to a delayed complication of a procedure I underwent in 2015.

On the eve of April 2022, while being on call during my three-month rotation in plastic surgery, I made my way back to the doctor's office after having managed a small laceration in the pediatric emergency department. As I sat down to catch a breather, I felt a slight discomfort in my lower abdomen, which I attributed to an occasional buildup of excess gas and assumed it would resolve spontaneously, which it did. However, it reappeared after a few minutes, with a slightly increased intensity this time around; nevertheless, it subsided on its own again. This pattern of appearance of the symptom followed by its self-resolution repeated a number of times, with the intensity and duration of the discomfort slightly increasing with each cycle, eventually turning into a persistent, dull ache in my right lower abdominal quadrant. I tried to carry out abdominal palpation on myself, albeit not in an ideal setting as I positioned myself supine on a crooked sofa. However, I was able to discern mild tenderness, particularly around the area of my appendectomy scar.

Toward the evening, I had started to feel unusually lethargic, and merely getting up from the office chair to go and attend to a patient had begun to feel like a Herculean task. A tachycardiac pulse directed me to the source of my extreme fatigue; I had developed a fever. Still, I ended up not seeking medical attention and continued to drag myself through the course of my duty hours that lasted until 2 PM of the following day, which in hindsight could have turned out to be a recipe for disaster.

**Figure 1:**
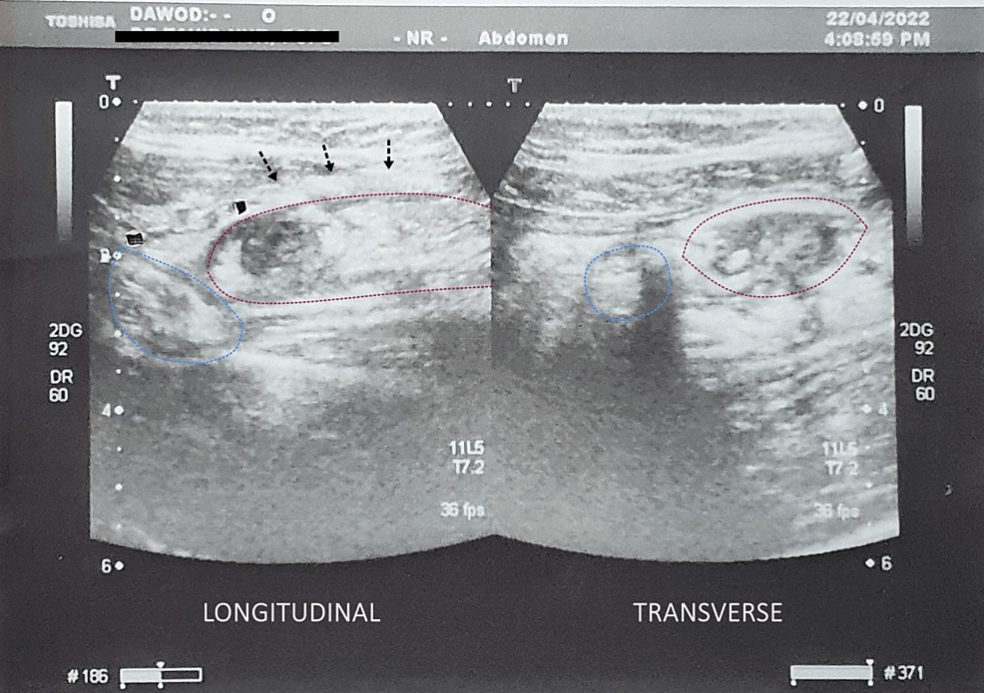
Initial abdominal ultrasound performed approximately 24 hours after the onset of symptoms. Longitudinal and transverse views reveal an edematous terminal ileum down to the ileocecal junction, a thickening of the ileocecal mesentery, and an inflamed appendicular stump. Dotted red line, terminal ileum; dotted blue line, appendicular stump; black arrows, ileocecal mesentery.

**Figure 2:**
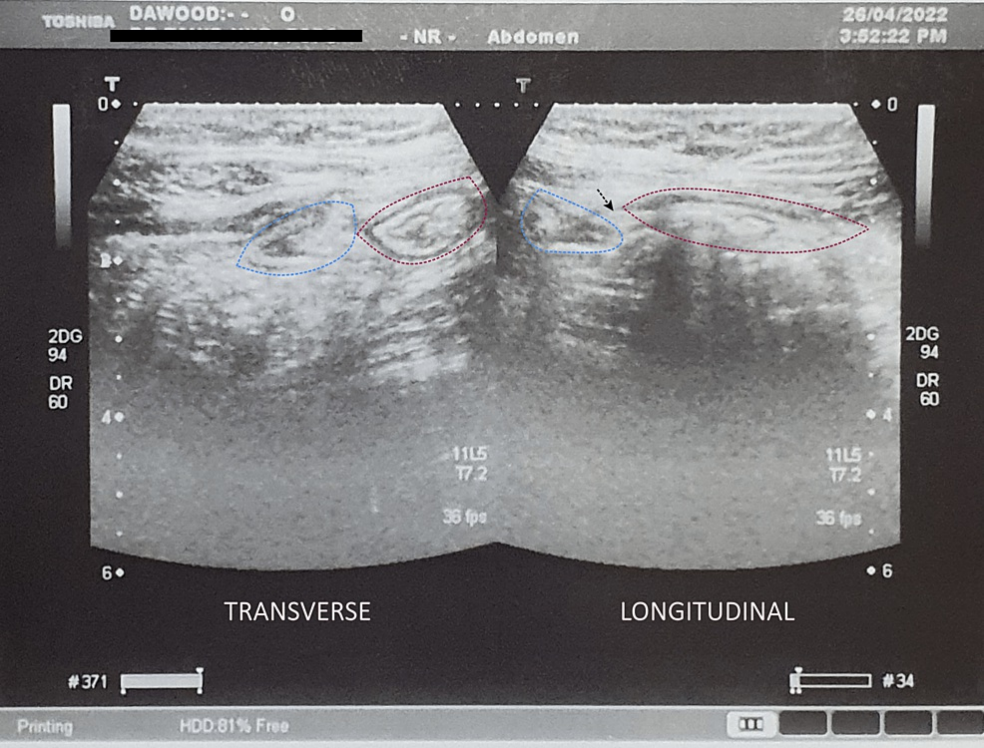
Abdominal ultrasound performed following a three-day course of intravenous antibiotics showing significant improvement. The terminal ileum is now mildly edematous and is encased by a thin streak of fluid. The appendicular stump, measuring 10 mm, is encased by a small focus of thickened mesentery. Dotted red line, terminal ileum; dotted blue line, appendicular stump; black arrow, thickened mesentery.

By the time I went back home, my symptoms had worsened, although I did not develop any additional ones. I narrated the entire history to my parents, both of whom are doctors themselves, and they were understandably concerned. My father, after examining me, immediately took me for an abdominal ultrasound to a consultant friend of his, who seemed to be slightly surprised by the discordance between the severity of symptoms and the nature of ultrasonographic findings, the latter revealinganedematous terminal ileum down to the ileocecal junction, a thickening of the ileocecal mesentery, and an inflamed appendicular stump (Figure 1). Later on, we consulted a gastroenterologist, another friend and former colleague of my father, who after inquiring about my detailed history and reviewing the ultrasound report considered stump appendicitis to be at the top of the possible differentials. I was advised baseline blood tests, although they peculiarly turned out to be within normal limits, and a three-day course of intravenous antibiotics, followed by a repeat abdominal ultrasound and a follow-up consultation. The post-antibiotic scan showed significant improvement, as evidenced by a reduction in the edema of the terminal ileum and a decrease in the inflammation of the appendicular stump, the length of which was reported as 10 mm* *(Figure 2). On the follow-up visit, I was advised oral antibiotics for an additional seven days, and after managing to make a complete recovery, I returned to the hospital for the resumption of my duties as a junior but a more learned doctor, taught by a host of lessons and the best of teachers: experience.

## Discussion

Epidemiology

Stump appendicitis, which results from the re-inflammation of the appendiceal stump, is an infrequent complication of a frequently performed surgical procedure, as evidenced by the reporting of a mere 164 cases until 2018 [3], beginning from the very first case documented by Rose in 1945 [4,5]. Consequently, a general lack of knowledge and understanding about this complication among physicians is often observed [6]. Furthermore, by virtue of its ability to mimic other causes of lower right abdominal pain, stump appendicitis is often not considered a possible differential of right iliac fossa discomfort, particularly if there is a past surgical history of appendectomy. However, whether the under-reporting of stump appendicitis in medical records is attributable to a low occurrence of the disease process itself or is due to its prowess to stay under the radar is debatable. The age group in which this complication presents is highly variable, as is the time interval from the index appendectomy procedure, with a reported range of 2-75 years and 2 weeks to 60 years, respectively [7].

The culprit factors

Various causes responsible for the development of this condition have been proposed, which can be broadly grouped into two categories: internal factors, relating to the elements at play within the body, and external factors, which mainly include the limitations of whichever surgical technique is employed. In either instance, the culprit factor is an inadequate identification of the appendico-cecal junction, resulting in a long appendiceal residue. The contributing internal factors include the obscuration of the field of view secondary to the presence of severe local inflammation; variants such as duplications, triplications [8], and appendiceal diverticula; the presence of multiple adhesions; and certain anatomical locations of the appendix, including retrocecal or subserosal [2]. Ensuring the appropriate identification of the appendiceal base and leaving behind a stump of not more than 5 mm have been proven to be effective in reducing the risk of this complication [9], although Roberts et al. suggest a maximal length of 3 mm [10].

With regard to external or surgical factors, few drawbacks associated with laparoscopic technique have been proposed to increase the risk of leaving behind a long appendiceal stump, such as a small two-dimensional field of view, the lack of haptic feedback, and poor depth perception. However, there is no concrete evidence to support the statement that the incidence of stump appendicitis increases with a laparoscopic approach. On the contrary, an extensive literature review conducted by Subramania and Liang revealed that 66% of the patients diagnosed with stump appendicitis were initially managed with an open technique [11]. Hence, it is suggested that it is the severity of the disease, more so than the surgical method, that dictates the subsequent incidence of stump appendicitis.

Investigations and treatment strategies

As the underlying process in both conditions is acute inflammation, the symptoms of acute appendicitis and stump appendicitis are similar, such as the classic right iliac fossa pain, fever, and gastrointestinal discomfort. The presence of such symptoms should prompt the clinician to consider stump appendicitis as a probable cause, even in the presence of an appendectomy scar. Two main investigation modalities are reported to be of assistance in diagnosing this condition: abdominal ultrasound and CT scan, although the latter is considered to be the gold standard, on account of its ability to exclude other etiologies of acute abdomen [12]. Nevertheless, some studies [13,14] vouch for the comparable effectiveness of ultrasonography as a diagnostic tool, and it may be considered as a preferable choice in instances where the treating physician is bound by time constraints, the lack of better facilities, and the unaffordability of the patient. Other reported investigative methods include diagnostic laparoscopy, colonoscopy, and barium enema, although these are rarely utilized. It is essential that the chosen investigation is swiftly employed, for a delay in diagnosis can lead to serious complications, including gangrene, intra-abdominal abscess formation, intestinal perforation leading to subsequent peritonitis, and intestinal obstruction secondary to adhesions.

Once the diagnosis of stump appendicitis has been established, prompt intervention is of the utmost importance. The usual course of treatment is via completion appendectomy, with a preference given to open technique, although a laparoscopic approach is also reported to have successful outcomes [15]. Rarely, severe inflammation around the ileocecal junction may be encountered, in which case extensive surgery such as ileocecal resection may be warranted [16]. However, nonsurgical management in the form of antibiotics has also proven to be equally effective in patients [3,7,17], including the case of the author himself, and may even be preferable owing to a lack of the usual risks and potential complications associated with surgical procedures.

Psychological implications and lessons learned

Attending to an excessively large number of patients, particularly in understaffed centers, doctors at times tend to look past the "human" aspect of an illness, merely treating the disease and not adopting a holistic approach while dealing with the patient, something which every health professional, including myself, can understand and relate to. However, as I was confined to a mattress in my room, scrolling aimlessly on my cellphone while waiting for the infusion to finish, I was able to appreciate the patients' perspective and the struggles they end up going through: the discomfort of getting your arm pricked multiple times for an intravenous access, the perturbing feeling of your entire body shivering uncontrollably minutes after the administration of an injection, not being able to bring yourself to eat a single bite of your favorite food even if it gets offered to you, helplessly watching everyone around you go about their daily routine while you are stuck in a situation where time seems to stand still, and feeling envious of your colleagues and peers as your mind constantly reminds you of everything you are missing out on; only after having gone through these experiences myself was I able to appreciate their gravity and the emotional toll they can take on a person. Another under-discussed factor that needs to be paid attention to is the impact a disease can have on a person's religious duties. Although religion is largely considered a private matter, it does hold a significant position in people's lives, particularly for those who are practicing. Thus, ill-health can be a central factor in disrupting a person's religious obligations, a fact I can substantiate, for I was unable to fast for a third of the holy month of Ramadan, an exceptionally sacred occasion for Muslims around the globe.

I was quite fortunate and perhaps privileged to have access to immediate and excellent medical care allowing me to make a swift recovery. Unfortunately, that is not the case for a majority of the public in Pakistan, who often end up bearing the brunt of an injudicious distribution of resources. Therefore, while it is important to introduce clinicians to this entity, it is equivalently essential to ensure the availability of medical facilities to every person, irrespective of their social or economic status. In addition, healthcare centers must also be supplied with state-of-the-art equipment so that the medical personnel can perform their duties efficiently, thus benefitting the patients in the long run.

Finally, this entire occurrence was reinforced by the belief in the adage "diseases do not read books." A fairly common disease can be encountered in the most unlikely manner, and a rare condition can have a textbook presentation. Although both the primary disease and its complication six years later manifested in an atypical manner in my case (on both occasions, I only experienced non-shifting abdominal pain and fever without any gastrointestinal symptoms), the clinical acumen of the professionals by whom I was treated put me on the path to recovery. Hence, every physician must strive for excellence, both in terms of knowledge and competence, rendering them able to tackle any clinical conundrum head-on. Having said that, it is equally crucial to understand when your body needs a break and to seek help if needed. Attempting to power through one's work at the expense of their physical and psychological health is not a rational idea, for it is bound to be unsuccessful and be a cause of detriment for everyone involved.

Limitations

This article is perhaps not unaffected by certain limitations, considering that the investigations and treatment modality employed in the author's case are not regarded as gold standard. In addition, as it is self-narrated, the possibility of the presence of some form of author bias cannot be entirely ruled out, although a maximally honest effort was made by the writer to ensure the article is devoid of any undesired elements.

## Conclusions

Stump appendicitis seems to slyly benefit from a vicious cycle, whereby a lack of information among healthcare professionals leads to it being underdiagnosed and consequently under-reported, which in turn further prevents it from being studied and recognized by medical specialists. While it is indeed a source of perplexity for the clinician, in essence, it is the patient who is affected, both physically and mentally. Thus, in addition to making clinicians more aware of this condition and encouraging them to adopt a multidimensional approach in clinical practice, there is a need for ensuring the meticulous execution of existing surgical techniques, along with the development of more advanced and refined methods in the future, as prevention is indeed the best cure.

The aforesaid discourse can be applicable to any condition, ranging from the extremely prevalent cases to the rarely encountered ones. However, due diligence and respect must be given to the latter, for these often end up severely affecting the patients' well-being. And while it is advisable to "think of horses when you hear hoofbeats," perhaps it is not too imprudent to consider zebras every once in a while.
